# UV-C Treatment Impact on the Availability of Water-Soluble Carbohydrates, Polyphenols, and Antioxidant Capacity of an Algerian Underutilized Date Fruit (*Phoenix dactylifera* L.)

**DOI:** 10.3390/foods13060893

**Published:** 2024-03-15

**Authors:** Kahina Djaoud, Rocío De la Peña-Armada, Alejandra García-Alonso, Virgilio Correcher, Lila Boulekbache-Makhlouf, Inmaculada Mateos-Aparicio

**Affiliations:** 1 Laboratoire de Biomathématiques, Biophysique, Biochimie, et Scientométrie, Faculté des Sciences de la Nature et de la Vie, Université de Bejaia, Bejaia 06000, Algeria; 2 Department of Nutrition and Food Science, Universidad Complutense de Madrid, 28040 Madrid, Spain.; alejandra.garcia.a@ucm.es (A.G.-A.); 3 Unidad de Dosimetría de Radiaciones, Departamento Medio Ambiente, Centro de Investigaciones Energéticas, Medioambientales y Tecnológicas, Av. Complutense, 40, 28040 Madrid, Spain; v.correcher@ciemat.es

**Keywords:** dates, UV-C radiation, carbohydrates, polyphenols, dietary fibers, techno-functional properties, antioxidant activity

## Abstract

Underutilized dates are considered as a socioeconomically important fruit for local and global communities, such as Degla-Beida, a common date fruit variety. The aim of this research was to elucidate, for the first time, the efficiency of UV-C light treatment (over different irradiation durations 5, 10, 20, and 40 min) in the enhancement of soluble carbohydrates and phenolic compounds, and to evaluate its effect on the antioxidant capacity. Furthermore, the content of dietary fiber was analyzed: insoluble dietary fiber (11.89 g/100 g); soluble dietary fiber (5.15 g/100 g); and total dietary fiber (17.06 g/100 g). The techno-functional properties were also determined: swelling capacity (3.94 mL/g); oil holding capacity (7.38 g/g); water holding capacity (9.30 g/g); and bulk density (1.81 g/mL). All were carried out to study the potential of exploiting this underutilized fruit for other applications as for feed or food. The results suggest that UV-C technology changes minimally the total water-soluble carbohydrate content; however, this preservation technology can affect the availability of different soluble carbohydrates depending on the irradiation time (IT), increasing the high molecular weight polysaccharides with IT up to 20 min, and some oligosaccharides with IT up to 5 min. The polyphenolic content determined by HPLC-QTOF was increased when the samples were submitted to UV-C reaching the maximum at 20 min (111.62 mg/100 g) and then to decrease in those submitted to IT of 40 min (12.05 mg/100 g). Regarding antioxidant capacity in the UV-C treated samples, FRAP decreased and EC50 on DPPH increased when IT was increased, while ORAC was hardly maintained. In addition, considering UV-C radiation associated with preservation and the studied date fruit as a rich source of dietary fiber with adequate techno-functional properties, this study presents valuable information for its potential use as a new food ingredient.

## 1. Introduction

Resource depletion and the inadequacy of waste management models pose major challenges for modern society, highlighting the need for a transition towards circular eco-nomic systems [1]. Creating effective strategies for the management and valorization of agri-food products constitutes one of the major global challenges [2].

The date palm (*Phoenix dactylifera* L.), a vital species in numerous Mediterranean regions, is often recognized by the widely commercialized Deglet Nour variety. Nonetheless, many other prevalent varieties, which could be of significant importance, often remain unknown and neglected [3]. Approximately 30% of dates end up being discarded or relegated to animal feed due to issues like undesirable texture (i.e., too hard or too soft), insect infestation, fungal contamination, or perceived low value and quality [4]. However, the use of such by-products for only limited purposes represents a significant economic loss, considering their substantial potential as valuable raw materials for extracting bioactive compounds and generating value-added resources [5]. The valorization of rejected second-grade date fruit to produce low-cost value-added products not only responds to environmental concerns, but also encourages industrial entities to promote a global perspective on the utilization and production of these ingredients [6].

Researchers have extensively studied the characterization and extraction of various high-value segments from second-grade dates [6]. Nutritionally, date fruits are an important source of carbohydrates (total sugars: 50–80 g/100 g dry weight (dw)) and dietary fiber (6.4–11.5 g/100 g dw, predominantly insoluble dietary fiber), with low levels of fats (0.2–0.5 g/100 g dw) and proteins (1.6–4.7 g/100 g dw) [7]. Furthermore, they contain essential micronutrients such as potassium, calcium, magnesium, sodium, manganese, zinc, and iron, as well as B-complex vitamins and vitamin C, along with various bioactive compounds including phenolic compounds, anthocyanins, sterols, and carotenoids, which contribute to their functional and health-promoting properties [8,9]. However, the composition of date fruits varies significantly depending on factors such as the ripening stage, cultivar, growing region, and climatic conditions [10]. As they progress through the ripening stages, date fruits undergo noticeable changes in color, texture, taste, and chemical composition [11]. At the final stage of ripening (tamar), the fruit reaches full maturity, with reduced moisture content, making it the best time for consumption [12].

Incorporating date fruit into a nutritious diet is advantageous [13]. Nevertheless, the lack of awareness of its benefits, especially among consumers, may be attributed to the prevailing trade practices in the developing date-producing countries. These nations export mainly dried dates to developed countries, which offer a long shelf life but lower nutritional value and higher sugar content [14]. To alter this pattern, collaboration between researchers and the food industry, along with their innovation and development teams, is essential. Their collective efforts can lead to the introduction of a wider range of nutritious date products into wider markets [12]. Thus, in addition to being consumed as dried or fresh dates (with local consumption), there should be a diverse range of date-based products available for commercial purposes, such as date juice, syrup, sugar, and dietary fiber [12]. The valorization of food by-products can be achieved through both conventional and environmentally friendly techniques. Conventional extraction methods often suffer from drawbacks like low yield, high cost, and the use of harsh chemicals, leading to environmental concerns and reduced effectiveness [15]. Therefore, it is crucial to adopt optimized recovery processes that involve pre-treatments and extraction methods [16]. UV-C treatment presents several advantages, including rapid microbial inactivation, minimal loss of flavor and nutrient, and lower energy consumption [17,18].

The United States Food and Drug Administration (FDA) and the European Food Safety Authority (EFSA) have approved UV-C light treatment as a valid methodology for controlling microorganisms in food products. When exposed to UV-C light, the DNA of pathogens is degraded, causing their disappearance by damaging cellular structures through the process of photo-oxidation [19]. Furthermore, EFSA declared the safety of UV-treated bread and milk as a novel food resulting in an increase in vitamin D concentrations [20]. Additionally, UV-C exposure induces the biosynthesis of phytoalexin compounds, bolstering product protection against subsequent infections [21].

Moreover, UV-C treatment enhances the activity of defense mechanism enzymes, effectively inhibiting bacterial growth [22,23]. Prior summaries on UV-C irradiation have primarily concentrated on its applications in water disinfection, preserving fruits and vegetables, sterilizing dairy products, and ensuring meat safety and quality [24,25,26]. Nevertheless, UV-C irradiation has been shown in prior food research to induce availability of phytochemicals in various fruits and vegetables. Specific studies have reported increased carbohydrate content after UV-C treatment [27,28], and also, showed maintenance, or even, higher levels of polyphenols and antioxidant capacity [29,30].

However, information on changes occurring to the components and properties of date fruit, after UV-C processing, is rather limited, prompting our characterization of these compounds at different UV-C treatment durations. UV-C treatment may affect the levels or properties of phytochemical compounds in date fruit. This will involve studying the potential impact of UV-C irradiation on the nutritional and bioactive compounds in date fruit, including polyphenols, poly-, oligo-, di-, and monosaccharides, and how changes in their composition or concentration may contribute to variations in antioxidant capacity or other relevant parameters. Herein, the present research aims to investigate the potential valorization of date fruits of low commercial value through the study of the effect of UV-C irradiation on the recovery of soluble carbohydrates, dietary fiber, and phenolic compounds. To the best of our knowledge, this is the first study exploring this work, providing thereby an additional insight into the valorization potential of low-grade (lost) date varieties, which could be used to develop novel functional food products.

## 2. Materials and Methods

### 2.1. Chemicals and Reagents

Analytical reagent grade chemicals were used. Ultrapure water (Milli-Q^®^ Advantage A10 Water Purification System from Millipore, Merck KGaA, Darmstadt, Germany) was employed for the mobile phase and other standard solution preparations. Pullulan 100 (100 kDa), Pullulan 50 (50 kDa), Pullullan 20 (20 kDa), and Pullulan 10 (10 kDa) from a Shodex Pullulan standard P-82 kit were acquired from Waters, Madrid, Spain. Inulin (5.94 kDa), verbascose (0.83 kDa), stachyose (0.67 kDa), cellotriose (0.50 kDa), rhamnose (0.50 kDa), glucose (0.18 kDa), and fructose (0.18 kDa) were acquired from Sigma, (Alcobendas, Madrid, Spain). Cellobiose (0.34 kDa) and sucrose (0.34 kDa) were acquired from Merck (Darmstadt, Germany).

### 2.2. Plant Material

Second-grade texturally defective (relatively hard) date fruits belonging to an Algerian variety, namely “Degla-Beida”, were collected from the oasis of Biskra (South of Algeria) at the ‘‘Tamr stage” (full ripeness) in the fall 2019 season. A total of 20 kg of Degla-Beida date fruit variety was obtained from a single batch to ensure uniformity and consistency in the experimental procedures. The dates were cleaned, pitted, dried at 40 °C to a constant weight, grounded into a fine powder with particle size ≤250 μm to minimize variability and facilitate uniform extraction and measurements, and stored [31]. The samples were transported to the laboratory of the Department of Nutrition and Food Science of Complutense University of Madrid in Spain for subsequent analyses.

### 2.3. UV-C Light Treatment

The UV-C irradiation experiments were conducted, in triplicate, to examine the effect of the treatment on the carbohydrate content before and after exposure of samples to UV-C radiation. They were performed on 10 g powdered aliquots by means of an automated irradiator developed at CIEMAT that allows UV illumination with a TUV-6W Hg lamp (254.7 nm, UV irradiance value at 10 cm, where the irradiance value, estimated to ensure uniform treatment across all samples, was 0.03 W·m^2^) [32]. Each of the 10 g sample batches (four batches), of date powder (DFP) were placed in transparent plastic bags that allow UV-C to pass through. Then, each batch was individually exposed to a UV-C irradiation time of for 5, 20, 30, and 40 min at retention time (RT); four irradiations in total. After the treatments, the samples were stored at 4 °C for 24 h before determinations of techno-functional properties, and the extraction of soluble carbohydrates and polyphenols. These extracts were analyzed by HPLC-RID for soluble carbohydrate composition (Section 2.6), by HPLC-QTOF for polyphenol profile (Section 2.7), and multifunctional antioxidant capacity was also measured (Section 2.8). All the measurements were made in triplicate.

### 2.4. Techno-Functional Properties

The water holding capacity (WHC) of date fruit powder (DFP) was determined according to the procedure set by Mateos-Aparicio et al. [33]. An amount of 250 mg of the sample was combined with 15 mL of distilled water in a 50 mL centrifuge tube. The mixture was shaken and left at ambient temperature for 1 h. After centrifugation at 3000× *g* over 20 min, the supernatant was removed, and the remaining residue was weighed. The WHC was determined by calculating the quantity of water held per each gram of dry sample. The initial dry weight of each sample was identified as W1, the weight after treatment was denoted as W2. The method for determining bulk density (BD) was adopted from Benítez et al. [34]. BD was assessed by filling a graduated cylinder with an equivalent volume of 5 mL of the sample. Subsequently, the cylinder was continuously tapped until a constant volume was obtained (V). The WHC and BD were then calculated as follows:

WHC = (W_1_ − W_2_)/W_1_ (1)

BD = W_1_/V (2)

The oil holding capacity (OHC) and swelling capacity (SWC) were measured by the procedure of Mateos-Aparicio et al. [33]. The identical procedure as described above for WHC was conducted, but instead of water, commercial virgin olive oil was utilized. OHC was quantified as the quantity of olive oil held per gram of dry sample. The 250 mg of sample was placed in a 10 mL measuring cylinder (with graduations of 0.1 mL) and mixed with 5 mL of distilled water containing 0.02% sodium azide. After gently stirring to remove trapped air bubbles, the mixture was left undisturbed on a flat support at room temperature overnight to allow for the sample to settle. Subsequently, the volume (in mL) taken up by the settled sample was recorded, and the suspended solids (SWC) were expressed as mL per gram of dry sample. The sample mass was noted as m_1_, and the discarded supernatant oil residue mass after centrifugation (8000 rpm for 10 min) was recorded as m_2_. The final volume after 24 h hydration at 4 °C was registered as V. The OHC and SWC were then calculated as follows:

OHC = (m_1_ − m_2_)/m_1_ (3)

SWC = V/m_1_ (4)

### 2.5. Characterization of Dietary Fiber

Date fruit powder (DFP) was analyzed for different dietary fiber fractions. The enzymatic–gravimetric method (AOAC 991.43) was employed to evaluate the total and insoluble dietary fiber (TDF and IDF) contents. Briefly, the samples (1 g) were incubated with heat-stable alpha amylase (100 °C, pH 6, 30 min), and then enzymatically treated with protease (60 °C, pH 7.5, 30 min), followed by incubation with amyloglucosidase (60 °C, pH 4.5, 30 min) to eliminate protein and starch. After that samples were filtered, washed with water, 95% ethanol, and acetone, then dried and weighed to quantify the insoluble fiber fraction (IDF). For the determination of total fiber fraction (TDF), four parts of 95% ethanol (400 mL at 60 °C) were added to the Erlenmeyer flasks containing the digesta samples with a ratio 4:1 *v*/*v*. Then, the pellets were filtered and washed subsequently with 78% ethanol, 95% ethanol, and finally with acetone. Later, the residues (total dietary fiber TDF) were dried and weighed. The ash and protein values determined in the residues were used to correct the fiber values. Soluble dietary fiber (SDF) content was calculated as the difference between TDF and IDF. The gravimetric residues were hydrolyzed, and the released neutral sugars transformed into alditol acetates to quantify by gas–liquid chromatography (GLC-FID) in a Perkin-Elmer Autosystem Chromatograph equipped with a hydrogen flame ionization detector. An SP-2330 column (30 m long, 0.25 mm i.d., and 0.25 μm film thickness) was used and nitrogen was the carrier gas. The injector and detector temperatures were 275 °C while the oven temperature was 235 °C. The uronic acid amount was determined in the hydrolysates following the colorimetric method of 3,5-dimethylphenol with Synergy™ HTX Multi-Mode Microplate Reader; galacturonic acid (Merck) was used as standard. The dietary fiber content was the sum of both neutral sugars and uronic acids. The results were given as g/100 g of dry weight [33].

### 2.6. Characterization of Soluble Carbohydrate Composition by HPLC-RID

A control amount of 250 mg and the UV-C light treated samples were weighed in a falcon tube and mixed with 10 mL distilled water. Samples were dissolved by agitation over 60 min, centrifuged, and filtered using 0.22 mm syringe filters. Date carbohydrates were analyzed with an HPLC system—Agilent 1200 series autosampler, Agilent quaternary pump system 1100 series with online degasser, Agilent 1100 series chromatography thermostatic oven, Agilent HPLC control unit 1100 series fitted with an Agilent 1100 series refractive index detector (RID), using an ionic exchange column RezexTM RSO-Oligosaccharide Ag 4%, LC Column (200 × 10 mm), preceded by a RezexTM RSO-Oligosaccharide Ag 4%, LC Guard Column (60 × 10 mm) (Phenomenex^®^, Torrance, CA, USA), with an isocratic mobile phase of HPLC grade water, retained at a flow rate 0.3 mL/min, and an injection volume of 5 μL at room temperature following the method from De la Peña-Armada et al. [35]. All the samples were analyzed in triplicate and data acquisition and analyses were carried out with an Agilent ChemStation from Agilent Technologies (Santa Clara, CA, USA) including identification by RT coincidence and quantification according to peak area, using the following equations: Pullulan 100 (y = 115.15x − 1799.9); Pullulan 20 (y = 137.85x − 4316); cellotriose (y = 115.51x − 909.27); saccharose (y = 132x + 982.05); glucose (y = 149.11x + 7428.1); fructose (y = 144.13x + 4927.4). The results were expressed in g/100 g dw.

### 2.7. Characterization of Phenolic Compounds by HPLC-QTOF

The HPLC (Agilent Technologies, Waldbroon, Germany) combined an integrated degasser with a quaternary pump, an autosampler, and a thermostatic column compartment. This unit was coupled to a hybrid mass spectrometer quadrupole-time of flight via an electrospray ionization source (ESI) with JetStream technology (Agilent Accurate Mass QTOF LC-MS, Waldbronn, Germany) and a diode array detector. The column used was a Zorbax Eclipse XDB C18 Agilent 150 mm × 5 μm × 4.6 mm. A binary system consisting of 0.1% formic acid in water (solvent A) and 0.1% acetonitrile in aqueous formic acid (solvent B) was used to obtain an elution gradient. The gradient applied was: 0 min, 95% (A), 5% (B); 20 min, 85% (A), 15% (B); 30 min, 70% (A), 30% (B); 35 min, 50% (A), 50% (B); 37 min, 95% (A), 5% (B) at a flow rate of 1 mL/min. The column temperature was 40 °C and the injection volume was 10 μL. Regarding, the quantification of the phenolic compounds calibration curves of commercial standards (Sigma, St. Louis, MO, USA) were employed, namely, chelidonic acid, vanillic acid (two different isomers), luteolin, syringic acid, trans cinnamic acid, isorhamnetin hexoxide, isorhamnetin hexoxide rhamnoside, quercetin hexoxide, luteolin rutenoside, kaempferol rutinoside, luteolin hexoxide, kaempferol hexoxide, quercetin derivative, catechin–epicatechin dimer, hydroxybenzoic acid, protocatechuic acid, gallic acid, caffeic acid, trans p-coumaric acid, rutin, quercetin, trans-ferulic acid, ferulic acid, chlorogenic acid, catechin, and cyanidin-glucoside.

### 2.8. Multifunctional Antioxidant Capacity

Date fruit extracts, both before and after UV-C treatment, were subjected to analysis for Fast-Blue, Folin–Ciocalteu, ferric reducing antioxidant power (FRAP), as well as oxygen radical absorbance capacity (ORAC) and DPPH-free radical scavenging activity on a Synergy HTX Multi-Mode microplate reader (Bio-Tek Instruments, Winooski, VT, USA).

The total phenolic content (TPC) of the sample extracts was assessed using two distinct assays. The Folin–Ciocalteu method was employed, following the protocol outlined by Singleton et al. [36]. Briefly, for the quantification of total phenolic content (TPC), Folin–Ciocalteu reagent (15 μL) (Scharlab, S.L., Sentmenat, Barcelona, Spain) was added to the samples (15 μL) and to gallic acid standards (0.01–0.30 mg/mL) (Sigma-Aldrich Química S.A, Madrid, Spain). Subsequently, 120 μL of distilled water and 30 μL of Na_2_CO_3_ were added to the samples and the standards, followed by another 120 μL of distilled water. A Synergy HTX Multi-Mode microplate reader (Bio-Tek Instruments, Winooski, VT, USA) at 750 nm was used to mesure the absorbance. Additionally, TPC was also determined by the Fast-Blue method reported as a novel technique by Medina [37]. In this method, 150 μL of gallic acid standards (0.01–0.30 mg/mL) and the samples were mixed with 15 μL of 0.1 g/100 mL Fast-Blue diazonium dye (Sigma-Aldrich Química S.A, Madrid, Spain), followed by 15 μL of 5 g/100 mL NaOH. After 90 min of reaction, the absorbance was measured using a Synergy™ HTX Multi-Mode microplate reader (Bio-Tek Instruments, Winooski, VT, USA) at 420 nm. Results were expressed as milligrams of gallic acid equivalents (GAE) per gram of dry weight (dw).

Underutilized date fruit extracts were evaluated for their antioxidant capacity through ferric reducing antioxidant power (FRAP), DPPH-free radical scavenging activity, and oxygen radical absorbance capacity (ORAC) assays. All measurements were conducted using a Synergy™ HTX Multi-Mode microplate reader (Bio-Tek Instruments, Winooski, VT, USA). For the FRAP assay, samples and 9 μL of Trolox standards (0.1–0.9 mmol/L) (Sigma-Aldrich Química S.A, Madrid, Spain) were combined with 265 μL of freshly prepared FRAP reagent and 26 μL of distilled water. The FRAP reagent was prepared using 2.5 mL of TPTZ (Sigma-Aldrich Química S.A, Madrid, Spain) solution at 10 mmol/L, 25 mL of acetic/acetate buffer (Scharlab, S.L., Sentmenat, Barcelona, Spain) at 0.3 mol/L, pH 3.6, and 2.5 mL of iron (III) chloride (Sigma-Aldrich Química S.A, Madrid, Spain) solution at 0.03 mol/L, warmed at 37 °C. Absorbance readings were taken at 595 nm after a 30-min incubation period, and results were expressed as millimoles of Trolox equivalents (TE) per gram of dry matter (mmol TE/g dw) [38]. ORAC assay was adapted from the method described by Serra et al. [39], using a microplate fluorescent reader (Synergy™ HTXMulti-Mode, BioTek, Winooski, VT, USA). Trolox standards, which concentrations ranged from 5 to 40 µM, and samples (12.5 µL) were pipetted into a 100 μL 96-well microplates. The peroxyl radicals (ROO) were generated from AAPH (Sigma-Aldrich Química S.A, Madrid, Spain) and the antioxidant scavenging ability was measured using disodium fluorescein included in the inner wells of the microplate (75 µL) (Merck, Darmstadt, Germany). The results of antioxidant capacity were expressed as micromoles of Trolox equivalents per gram of dry matter (μmol TE/g dw). The DPPH assessment followed the methodology described by Karadag et al. [40]. Briefly, 20 μL of Trolox (Sigma-Aldrich Química S.A, Madrid, Spain) standards prepared in ethanol 80% (0.01–0.9 mmol/L) and samples were mixed with 280 μL of 100 μmol/L DPPH solution (Sigma-Aldrich Química S.A, Madrid, Spain). After a 30-min incubation period, absorbance was measured at 517 nm, and was calculated. Results of the free radical scavenging activity were reported as EC50 (the concentration required to achieve a 50% antioxidant effect).

### 2.9. Statistical Analysis

The data, expressed as mean values ± standard deviation, were subjected to analysis of variance (ANOVA), Tukey’s test at 5% significance, and principal component analysis (PCA) was performed using the XLSTAT Release 10 (Addinsoft, Paris, France).

## 3. Results and Discussion

### 3.1. Techno-Functional Properties

The properties of food products reflect their essential physicochemical characteristics based on interactions between structures and molecules in different compositions of ingredients. Therefore, evaluating the techno-functional properties may help to predict the ingredient behavior in specific food systems, and thus, the quality of the food [41].

Swelling capacity (SWC) is a parameter usually applied to measure the hydration capacity. It is the volume in milliliters taken up by the swelling of one gram of food material [42]. The water retention capacity (WHC) is economically an important step to be validated by the food, cosmetic, and nutraceutical industries. The organoleptic and the texture of end food products are strongly related to the WHC of its components. This is generally based on the interactions between proteins and water [43]. Analyzed date fruit sample showed SWC and WHC values of 3.943 ± 0.003 mL/g and 9.30 ± 0.25 g/g, respectively (Table 1). The observed values suggest a notable correlation between the two properties. This relationship is indicative of the sample’s efficiency in absorbing and retaining water. These outcomes fell within the range observed by Teye et al. [44] (3.2–4.5 mL/g) in sweet potato flour and some underutilized seasonal vegetables, surpassing those found by Jose et al. [45] (0.16–0.26 mL/g) and Lorente et al. [46] (2.34–2.97 mL/g) in pineapple pomace and Turrón coproducts, respectively, and remaining below those indicated by Martínez et al. [47] (4.6–11.4 mL/g) in clementine by-products for swelling capacity. Regarding water retention capacity, the finding aligns with Martínez et al. [47] with the range 6.00–10.60 g/g in clementine by-products but stands higher than those reported by Teye et al. [44] (1.42–1.96 g/g) in sweet potato flour and some underutilized seasonal vegetables, Jose et al. [45] (5.15–5.69 g/g) in pineapple pomace, Megías et al. [48] (2.4–21.3 g/g) in grape pomace, Alvarez et al. [49] (1.91–4.47 g/g) in grape seed flour, and Lorente et al. [46] (4.11–4.97 g/g) in Turrón coproducts.

The oil holding capacity (OHC) is expressed as the amount of oil absorbed per gram of product, and it depends mainly on protein conformation, amino acid composition, and surface hydrophobicity. The ability of an ingredient to absorb oil can influence the organoleptic and textural properties, enhancing the flavor and mouthfeel of the final product [41,50]. The date fruit sample showed an OHC value of 7.38 ± 0.30 g/g and a bulk density of 1.81 ± 0.01 g/mL (Table 1). This outcome exceeded the values reported by Lorente et al. [46] (1.81- 2.08 g/g) in Turrón coproducts, Jose et al. [45] (2.38–2.76 g/g) in pineapple pomace, Martínez et al. [47] (2.1–2.6 g/g) and Alvarez et al. [49] (1.94–4.67 g/g) for OAC in clementine by-products and grape seed flour, respectively. However, it was lower than those found by Megías et al. [48] (14.1–25.4 g/g) in grape pomace. The bulk density is important in determining the packaging requirement, material handling, and application in processing in the food industry [51]. Low bulk density is a desirable factor in food formulation especially for food with less retrogradation [52]. However, high bulk density is a good physical attribute when determining the mixing quality of a particular matter [51]. A lower bulk density suggests a relatively porous structure. This porosity enhances the oil holding capacity by providing an increased surface area and interstitial spaces for oil absorption. The combination of high oil adsorption capacity and low bulk density in date residues opens up innovative possibilities in the food sector. Leveraging these properties can lead to healthier, more sustainable, and technologically advanced food products. Further research in this direction holds the potential to contribute to the development of effective and eco-friendly solutions. The data suggest the possibility to use these by-products as a functional food ingredient, to reduce calories, to avoid syneresis, to stabilize high fat food products, and as a modifier of viscosity and texture of some formulated foods; this is probably due to the ability of dietary fibers to absorb water [53]. Dietary fiber, both insoluble and soluble fractions, and other carbohydrates influence the food techno-functional characteristics. UV-C treatment caused a rupture in cellular membranes that would diminish cell compartmentalization and could affect the solubility of components including carbohydrates, which may improve the techno-functional properties. Likewise, greater quantities of phenolics may contribute antioxidant mechanisms during food storage with heating conditions to resist nutritional losses as well as provide binding interactions to improve texture. Elucidating such structure–function contributions of impacted components will be imperative in confirming if postharvest UV-C exposure can tailor dates for sustainable industrial applications.

### 3.2. Characterization of Dietary Fiber Composition

As has been widely shown in the literature, the main components of date fruit (*Phoenix dactylifera* L.) are dietary fiber and sugars, which depend mostly on the variety, climatic variations, and geographical origins, and less on the seasonal, processing, and storage conditions [54]. Accurate determination of total dietary fiber (TDF) and its soluble (SDF) and insoluble (IDF) fractions is relevant for food analysis research and nutritional labeling [55]. A gas–liquid chromatographic method (GLC) was developed for the separation and quantitative determination of neutral sugars. Sample preparation involved enzymatic removal of starch and acid hydrolysis of the non-starch polysaccharides to their constituent sugars [56]. Monosaccharides commonly present in plant cell walls are the following: (1) hexoses—D-glucose, D-galactose, D-mannose; (2) pentoses—L-arabinose, L-xylose; (3) 6-deoxyhexoses—L-rhamnose, L-fucose; (4) uronic acids—glucuronic and galacturonic acids [57].

Allose was chosen as an internal standard (IS) since it was eluted within the time that all sugars were eluted, and it did not co-elute with any of the tested sugars [55]. Here, in this study we outline the dietary fiber composition of Degla-Beida date fruit powder, as summarized in Table 2. The amounts of neutral sugars (principally formed by rhamnose, fucose, arabinose, xylose, mannose, galactose, and glucose), and uronic acids, were present in both total, insoluble, and soluble fractions (17.06 ± 0.25, 11.89 ± 0.25, and 5.15 ± 0.33 g/100 g), respectively. In the insoluble fraction, the predominant neutral sugars were arabinose (4.28 ± 0.10 g/100 g), xylose (3.17 ± 0.14 g/100 g), and galactose (1.03 ± 0.03 g/100 g). This indicates the presence of arabinoxylans and other hemicelluloses. It is remarkable that glucose is not the most important sugar in IDF when usually cellulose is the main polymer of this fraction. The main neutral sugars of SDF include rhamnose (1.39 ± 0.09 g/100 g), arabinose (0.79 ± 0.11 g/100 g), and uronic acids (0.71 ± 0.07 g/100 g) meaning the rhamnogalacturonan backbone probably substituted by arabinans. The soluble fraction typically contains sugars that are more easily dissolved in water, suggesting potential bioavailability and health benefits associated with these compounds. Considering the overall composition in the total fraction, the major neutral sugars are arabinose (5.07 ± 0.13 g/100 g), xylose (3.73 ± 0.13 g/100 g), and uronic acids (2.19 ± 0.10 g/100 g) indicating that hemicellulosic and pectic polysaccharides, such as arabinoxylans and rhamnogalacturonan backbone probably substituted by arabinans, are the main components in the cell wall. It is consistent with Noorbakhsh and Khorasgani [58] who describe the date fruit polysaccharides as mainly consisting of the pectic compound and (1→3)-β-D-glucopyranosyl residues. Several research studies strongly confirm high dietary fiber content in dates, showing that date products are a better source of dietary fiber than some cereal products such as rice and oats, and could be incorporated as an ingredient in fiber-enriched food [10,59,60]. The obtained data were higher than those reported by Bano et al. [61], oscillating between 5.48 to 9.83% for TDF, between 3.43 to 3.59% for SDF, and between 5.98 to 6.24% for IDF, while being lower than those recorded by Hashim and Khalil [54] ranging from 50.81 to 56.52% for TDF, from 6.19 to 9.15% for SDF, and from 41.66 to 49.99% for IDF.

The differences can be attributed to growth, climatic conditions, geographic origin, or the extraction methods, which are very important factors. Fibers are desirable in the food sector because they offer gut-health promotion via bulking effects and production of short-chain fatty acids [62]. Dietary fiber is widely recognized for its key role in functional attributes, in particular its ability to lower cholesterol levels. Worldwide, cardiovascular disease (CVD) is the leading cause of mortality, mainly due to the accumulation of fat in blood vessels, largely attributed to high levels of low-density lipoprotein (LDLs). Furthermore, dietary fiber serves as a valuable component in food product development, contributing to functions like gel formation and emulsification effects [10].

### 3.3. Characterization of Carbohydrate Content by HPLC-RID

**Figure 1:**
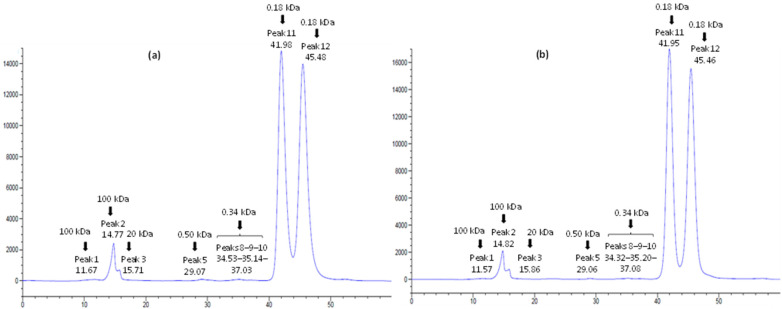
HPLC-RID analysis of water-soluble carbohydrates from untreated (**a**) and treated (**b**) date fruit powder during 20 min of irradiation with UV-C light.

In the current investigation, the total soluble carbohydrate content of Degla-Beida date fruit was recorded as 80.02 ± 0.01 g/100 g in the untreated sample, 80.03 ± 0.01, 80.05 ± 0.04, 81.10 ± 0.01, and 78.45 ± 0.01 g/100 g in the irradiated samples for 5, 10, 20, and 40 min of irradiation, respectively. The results presented in Table 3, derived from ionic-exchange HPLC-RID analysis of water-soluble carbohydrates, offer significant insights into how UV-C treatment affects the composition of the sample. A total of twelve peaks were discerned in the chromatogram profile (Figure 1). Quantification standards were set by comparing the proximity of each peak with the standard retention time. The peaks were designated as follows: peaks 1 and 2 as high molecular weight polysaccharides (quantified with the Pullulan 100 equation), peak 3 as a medium molecular weight polysaccharide (quantified with Pullulan 20 equation), peaks 4–6 as trisaccharides (quantified with the cellotriose equation), peaks 7–10 as disaccharides (quantified with the saccharose equation), peak 11 as glucose, and peak 12 as fructose. The main carbohydrates in the analyzed untreated and UV-C-treated samples are those corresponding to peaks 12 and 11, named fructose and glucose, respectively, as well as the carbohydrate from peak 2 corresponding to a high molecular weight polysaccharide (HMWP). The fructose (peak 12) amount was mainly maintained after UV-C treatments; however, glucose (peak 11) decreased slightly, and the peak around 100 KDa, polysaccharide (peak 2), increased with irradiation time (IT). This increase was also seen in peak 9 and 10. Peak 1 decreased with 5 and 10 min of irradiation, however, after 20 min increased. In addition, peak 3, corresponding to medium molecular weight polysaccharide, appeared in the treated samples (non-detected in the untreated one) except for the 40 min-irradiated sample. Peak 5 presented a similar behavior, meaning that this trisaccharide increased with the IT but not for 40 min, in which similar amounts as the untreated sample were detected. Peaks 6 and 7 only appear in the short time irradiated samples. Peak 4 was only observed in the 40 min-irradiated samples. Summarizing, although the total soluble carbohydrate amount was maintained, UV-C treatment could disrupt cellular membranes, solubilizing, modifying, or causing breakdown of certain carbohydrates, addressing the variations in their structural integrity.

The obtained results for the studied variety were higher than those noted by Abdul-Hamid et al. [14] (59.6‒76.8 g/100 g) in nine Saudi date palm fruit varieties and lower than those found previously by Djaoudene et al. [63] (84.51 to 96.28 g/100 g) for eight Algerian cultivars of date palm fruits. However, they were in the same range as those shown by Hariri et al. [64] (61.9‒85.7 g/100 g) for half-soft date varieties cultivated in Adrar (south of Algeria). Assirey [65] carried out an analysis of the total carbohydrate content in ten date varieties highlighting Burni (81.4 g/100 g) as the highest, followed by Suqaey (79.7 g/100 g) and Khodari (79.4 g/100 g), while Labanah recorded the lowest value (71.2 g/100 g). The amounts of the analyzed date variety quantified in our study (Table 3) were similar to those reported in some date fruit varieties grown in Pakistan [66]. But higher amounts for fructose (47.50 g/100 g), glucose (51.80 g/100 g), and sucrose (3.20 g/100 g) were reported by Abdul-Hamid et al. [14]. Taha et al. [67] found similar results for glucose content (26.50–42.30 g/100 g), lower for fructose content (14.98 to 22.50 g/100 g), and higher for sucrose content (6.17–7.46 g/100 g) in Deglet Nour variety from different oases in Tunisia. The fructose and glucose values obtained in the present study were higher than those determined in nine varieties of Algerian date palm fruits as reported by Hussain et al. [68] in their sugar profiling study (16.36‒16.60 and 17.38‒17.75 g/100 g, respectively).

The increased levels of glucose and fructose could be linked to the activity of the invertase enzyme, responsible for converting sucrose into glucose and fructose, a process that usually intensifies as the fruit ripens [69]. Variations in sugar concentration are likely to be influenced by environmental and genetic factors, impacting both the qualitative and quantitative composition of the sugar fraction through their effects on enzyme activity involved in synthesis, breakdown processes, and fruit maturation [61].

### 3.4. Characterization of Phenolic Compounds by HPLC-QTOF

**Figure 2:**
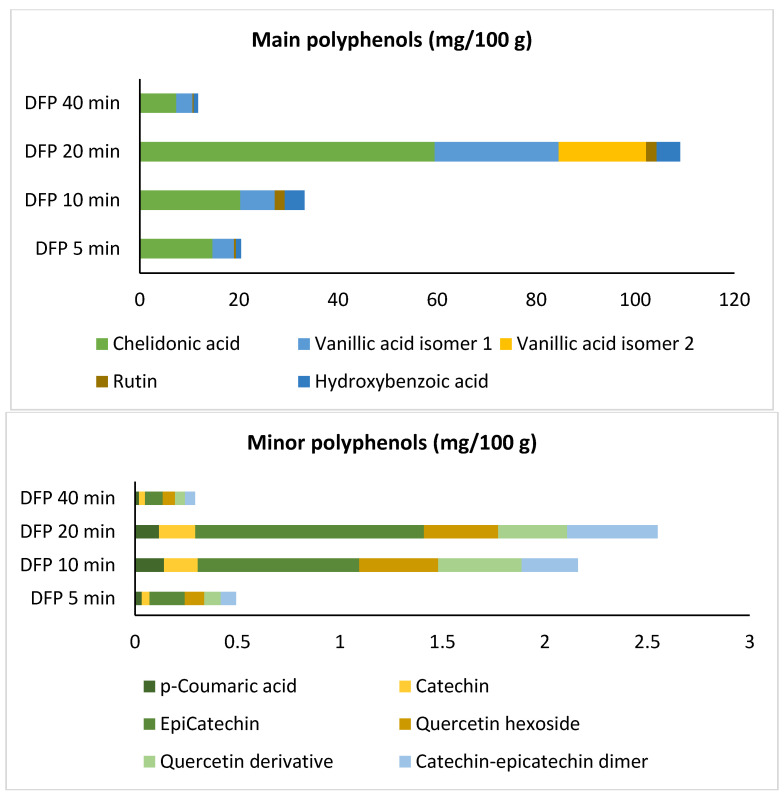
Main and minor polyphenols from date fruit powder, treated with UV-C irradiation for 5, 10, 20, and 40 min.

Phenolic compounds of date fruit have been extensively studied. Regarding the identification and quantification of phenolic acids and flavonoids, Airouyuwa et al. [70] identified ten polyphenols, namely, in descending order, 3,4-dihydroxybenzoic acid, caffeic acid, p-coumaric acid, ferulic acid, vanillic acid, rutin hydrate, catechin, cinnamic acid, 4-hydroxybenzoic acid, and syringic acid. Bouhlali et al. [71], as well, reported ten phenolic compounds, in descending order, p-coumaric acid, caffeic acid, rutin, quercetin, gallic acid, vanillic acid, luteolin, chlorogenic acid, ferulic acid, and syringic acid. Specifically, in the Degla-Beida variety [72], four phenolic acids (gallic, ferulic, p-coumaric, and caffeic) and five flavonoids (quercetin hexoside, rutin, isoquercetin hexoside, quercetin, and luteolin) were quantified. On the other hand, to the best of our knowledge, the current study investigated, for the first time, the phenolic composition of UV-C treated Algerian date fruit of the Degla-Beida variety. The polyphenol profile of irradiated date fruit polyphenols, performed by an HPLC-QTOF are presented in Figure 2. The polyphenolic content increased according to the duration of the UV-C treatment up to 20 min. However, the samples subjected to 40 min of treatment showed a decrease of this fraction. The main identified compounds for all the samples were chelidonic acid, followed by vanillic isomer 1. Chelidonic acid was reported earlier by Khallouki et al. [73] and Hilary et al. [74] in date fruit as the greatest of the phenolic acids. After UV-C irradiation, chelidonic acid decreased compared with date fruit [73] but at 20 min, it was found in a much higher amount. Vanillic isomer 2 was only identified in the date fruit powder subjected to 20 min of UV-C light. Rutin and hydroxybenzoic acid were found also among the major polyphenols identified. The greatest increase occurred after 20 min of treatment, from 20.97 to 111.62 mg/100 g, while a decrease was very remarkable after 20 min of UV-C light application, to achieve 12.05 mg/100 g at 40 min. Vanillic acid and hydroxybenzoic acid were found to be greater in all irradiated samples in comparison with date fruit [73]. Rutin was in a higher amount in all irradiated samples than in the Degla-Beida date [72] and only greater in 10- and 20-min irradiated samples than the Medjool date [73]. Results for the minor polyphenols identified in date fruit powder revealed that for epicatechin and quercetin derivative compounds, the rise was proportional to the duration of the treatment, while for the other analyzed polyphenols, namely p-coumaric acid, catechin, quercetin hexoxide, and catechin–epicatechin dimer, there were no considerable differences between the applications at 10 or 20 min of UV-C treatment (Figure 2). The irradiation seems to diminish p-coumaric acid, and to maintain similar amounts of quercetin and derivatives compared to date fruit [72,73]. Furthermore, the irradiated samples did not present ferulic and gallic acids, usually detected in date fruits [71,72,73]. The abundance and composition of phenolic compounds in *Phoenix dactylifera* subspecies changes greatly within cultivars, genetic characteristics, geographic locations, climatic conditions in the growing environment, and the extraction methods [3,70,71,72,73].

### 3.5. Antioxidant Capacity In Vitro

Dates offer a potential solution to relieve oxidative stress, a condition resulting from excessive production of free radicals, which contributes to the onset and progression of various diseases [7,75]. The presence of bioactive compounds in dates has attracted considerable attention from researchers, positioning this fruit as a promising natural source of antioxidants and functional food ingredients [76]. Additionally, studies have explored the effectiveness of UV-C irradiation in improving the quality and extending the shelf life of fruits and vegetables [2,77,78,79]. However, there is currently little information on the potential application of UV-C rays in the processing of second-grade dates. In this study, we evaluated the total polyphenol content (TPC) using Fast-blue and Folin assays and examined the antioxidant capacity of date powder before and after exposure to UV-C irradiation (for durations of 5, 10, 20, and 40 min) via FRAP, ORAC, and DPPH EC50 tests, as detailed in Table 4.

TPC reported by the Fast-Blue (FB) and Folin–Ciocalteu (F-C) methods, which estimate total phenolics based on redox reactions, was higher compared to total polyphenols from HPLC-QTOF (5 min—20.97 mg/100 g; 10 min—35.42 mg/100 g; 20 min—11.62 mg/100 g; 40 min—12.05 mg/100 g) (Section 3.3). The chromatographic techniques quantify individual phenolic compounds, so do not capture the total phenolic pools. However, the FB and F-C methods quantify TPC, based on an antioxidant mechanism. It is known that not all polyphenols develop the same antioxidant capacity, so this capacity is influenced by the type of polyphenols, meaning that a major polyphenol content is not necessarily always related to a major antioxidant capacity. Moreover, these spectrophotometric methods may have been influenced by non-phenolic reducing agents within the complex plant extracts analyzed. This phenomenon has occurred with other authors such as Benmeddour et al. (2013) [72] for Degla-Beida date, and other date varieties such as Khalloki et al. (2018) [73] for Medjool date.

As it is observed in Table 4, TPC and the antioxidant capacity (FRAP, ORAC, DPPH EC50) of date fruit exhibit a decline following UV-C irradiation, but after 40 min of UV-C treatment, that TPC (Fast-Blue and Folin) and ORAC were statistically similar to the non-treated samples. It is worth noting that exposing the samples to UV-C radiation for higher times resulted in the maintenance of ORAC and DPPH EC50 compared with non-treated samples, or in an increase if the comparison is done among irradiated samples.

As mentioned, there is not a similitude with the polyphenolic profile from HPLC-QTOF analysis (Section 3.3). As described, the polyphenol total amount was increased up 20 min to decrease at 40 min for similar contents of untreated samples, so similar results were expected for their antioxidant capacity. However, in the case of TPC (Folin and Fast-Blue), the results were similar in the untreated sample and the 40 min treated one, but this was because the polyphenols decrease from the 20 min to 40 min treated samples. Regarding ORAC, it dropped in the treated samples although there was not a significant difference (*p* > 0.05) in those treated after 5 and 40 min. Although a major antioxidant capacity was expected in those samples with more polyphenol content from HPLC analysis (samples treated with 20 min of UV-C irradiation), it did not occur.

Gamma irradiation can increase TPC and antioxidant capacity in almond skin extract, peanut skin, curcuma, and others [80,81,82]. On the other hand, it has been observed that this ionizing radiation could lead to a decrease in the antioxidant capacity of green tea, yerba mate, and chamomile tea [83]. However, changes in phenolics, whether by increase, decrease, or maintenance, are assumed to be because of molecular conversion, depolymerization, and cross-linking [81]. As UV-C is a partially ionizing radiation, it is assumed to have a less stimulating effect than gamma radiation on antioxidant capacity. Indeed, a decrease in the antioxidant capacity of horchata beverage treated with UV-C radiation against DPPH radicals was reported [84]. However, it was proved that UV-C can increase antioxidant capacity in fresh-cut mangoes, grape, and also in a by-product such as pineapple skin [85,86,87]. In fact, in our study a greater presence and/or reorganization of polyphenols was observed (Section 3.3), as expected, but this did not correspond to an increase in antioxidant capacity. This increased presence of polyphenols could be due to the mechanisms explained in previous studies through molecular conversion, depolymerization, and cross-linking with other substances. This interaction with other substances, mainly with polysaccharides, vitamins C/E, and carotenoids, causes frequently more potent antioxidant effects than those of the individual substances [88,89,90,91,92]. However, we postulate a possible competition between these polyphenols instead of a synergy to explain the lower antioxidant capacity compared to that which we expected. Polyphenolic compounds exhibit their respective antioxidant effects, but individual phenols cannot be easily isolated, and multiple polyphenols often exert synergistic or antagonistic antioxidant effects.

### 3.6. Principal Component Analysis (PCA)

**Figure 3:**
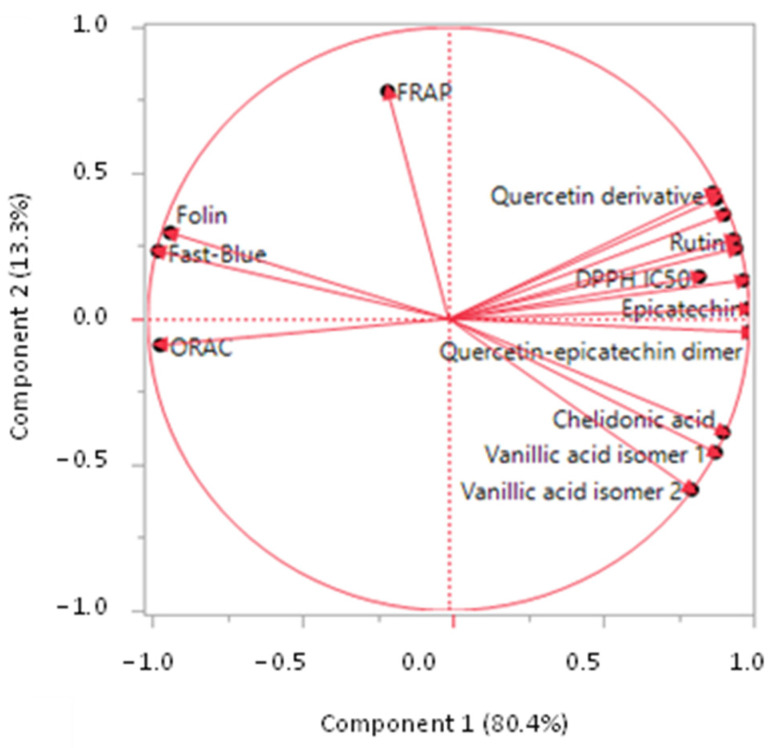
Principal components analysis (PCA) showing the correlation between phenolic compounds and antioxidant activity of date fruit powder treated with UV-C irradiation for 5, 10, 20, and 40 min.

Principal component analysis (PCA) was used for multivariate analysis of date fruit phenolic compounds and antioxidant capacity following UV-C irradiation for durations of 5, 10, 20, and 40 min, as shown in Figure 3. Principal Component 1 (PC1) accounted for 80.4% of the total variance, while PC2 explained 13.3%, collectively explaining 93.7% of the variability in the experimental data. Variables positioned closely together indicate correlation, with variables on the same side of the origin (0,0) indicating positive correlation, and those on opposite sides indicating negative correlation. Accordingly, all phenolic compound values (such as chelidonic acid, vanillic acid isomer 1, vanillic acid isomer 2, rutin, hydroxybenzoic acid, p-coumaric acid, catechin, epicatechin, quercetin hexoxide, quercetin derivative, and quercetin–epicatechin dimer) clustered on the right side of the plot, imply a positive correlation. Conversely, antioxidant capacity variables (Fast-Blue, Folin, FRAP, ORAC, and DPPH EC50) clustered on the left side, indicate a positive correlation among themselves. Variables on the right side (phenolic compounds) were negatively correlated with those on the left side (antioxidant capacity), except for the DPPH assay, which was expressed as EC50. Thus, PCA validates the previous observation that an increase in phenolic content may not necessarily correlate with the presence of active antioxidants. Structural alterations and the generation of new phenolic compounds induced by UV-C treatment may result in derivatives with reduced antioxidant efficacy. Phenolic compounds can engage in intricate interactions with other components in date powder, which may be influenced by UV-C treatment, potentially forming complexes that impede the antioxidant capacity of phenolic compounds. UV-C treatment might also modify the bioavailability of phenolic compounds, impacting their antioxidant potential. Changes in solubility or accessibility of phenolic compounds due to treatment could further affect their overall antioxidant capacity. In certain instances, UV-C exposure may induce pro-oxidant effects, triggering photooxidative reactions that could counteract the antioxidant properties of phenolic compounds.

## 4. Conclusions

This investigation provides the first attempt to elucidate the effect of UV-C exposure on the water-soluble carbohydrates, polyphenols, and antioxidant capacity of the Algerian underutilized Degla-Beida date fruit, which is found to be a rich source of dietary fiber, exhibiting adequate techno-functional properties. The UV-C treatment applied to this fruit has the potential to influence the availability of different water-soluble carbohydrates depending on the irradiation time (IT), increasing the high molecular weight polysaccharides with IT up to 20 min, and some oligosaccharides with IT up to 5 min. The polyphenolic content (HPLC-QTOF) increased when the samples were submitted to UV-C, reaching the maximum at 20 min (111.62 mg/100 g) and to decrease in those submitted to IT of 40 min (12.05 mg/100 g). Regarding antioxidant capacity in the UV-C treated samples, FRAP decreased and EC50 on DPPH was raised when IT was increased, while ORAC was hardly maintained. Thus, some polyphenols could be positively affected with UV-C irradiation, but it is not necessarily related to an increase of their antioxidant capacities. Considering the positive effect of UV-C radiation associated with preservation, it might open up a new horizon for further valorization of underutilized date by-products and their applications in food/feed. This highlights the interest of utilizing UV-C as a potential tool not only for preservation, but also for selectively enhancing or maintaining these compounds.

## Figures and Tables

**Table 1:** Techno-functional properties of date fruit powder.

Property	Value
Swelling capacity (mL/g)	3.94 ± 0.003
Oil holding capacity (g/g)	7.38 ± 0.30
Water holding capacity (g/g)	9.30 ± 0.25
Bulk density (g/mL)	1.81 ± 0.01

Data are mean values ± standard deviation.

**Table 2:** Insoluble dietary fiber (IDF), soluble dietary fiber (SDF), and total dietary fiber (TDF) of date fruit powder.

Monomers	Date Fruit Powder (DFP)		
	IDF (g/100 g)	SDF (g/100 g)	TDF (g/100 g)
Rhamnose	0.60 ± 0.05 ^de^	0.71 ± 0.07 ^bc^	1.31 ± 0.03 ^d^
Fucose	0.55 ± 0.03 ^e^	0.67 ± 0.03 ^c^	1.22 ± 0.03 ^e^
Arabinose	4.28 ± 0.10 ^a^	0.79 ± 0.11 ^b^	5.07 ± 0.13 ^a^
Xylose	3.17 ± 0.14 ^b^	0.56 ± 0.10 ^d^	3.73 ± 0.13 ^b^
Mannose	0.64 ± 0.05 ^de^	0.51 ± 0.14 ^de^	1.15 ± 0.13 ^ab^
Galactose	1.03 ± 0.03 ^c^	0.36 ± 0.11 ^e^	1.38 ± 0.10 ^d^
Glucose	0.98 ± 0.06 ^cd^	0.17 ± 0.03 ^f^	1.02 ± 0.09 ^f^
Uronic acids	0.78 ± 0.05 ^d^	1.39 ± 0.09 ^a^	2.19 ± 0.10 ^c^
Total	11.89 ± 0.25	5.15 ± 0.33	17.06 ± 0.25

Data are mean values ± standard deviation. Values with letters (a–f) were significantly different (Tukey, *p* < 0.05).

**Table 3:** Characterization of water-soluble carbohydrates from untreated and UVC-irradiated date fruit, for 5, 10, 20, and 40 min, by HPLC-RID.

Peak N°	Standards		Untreated (g/100 g)	5 min (g/100 g)	10 min (g/100 g)	20 min (g/100 g)	40 min (g/100 g)
	Mw (kDa)	RT (min)					
Peak 1	100	11.90	0.38 ± 0.03 ^b^	0.22 ± 0.03 ^c^	0.28 ± 0.01 ^c^	0.53 ± 0.05 ^a^	0.53 ± 0.02 ^a^
Peak 2	100	14.55	4.43 ± 0.08 ^b^	4.52 ± 0.02 ^a^	4.52 ± 0.02 ^a^	4.56 ± 0.03 ^a^	4.48 ± 0.04 ^ab^
Peak 3	20	15.20	nd	0.84 ± 0.03 ^c^	0.94 ± 0.03 ^b^	1.08 ± 0.03 ^a^	nd
Peak 4	20	15.85	nd	nd	nd	nd	1.32 ± 0.01 ^b^
Peak 5	0.50	29.00	0.17 ± 0.01 ^c^	0.42 ± 0.02 ^a^	0.37 ± 0.03 ^a^	0.33 ± 0.07 ^b^	0.16 ± 0.02 ^c^
Peak 6	0.50	30.00	nd	0.23 ± 0.04 ^a^	0.16 ± 0.02 ^b^	nd	nd
Peak 7	0.34	33.80	nd	0.05 ± 0.02 ^a^	nd	nd	nd
Peak 8	0.34	34.30	0.06 ± 0.01 ^a^	nd	0.03 ± 0.01 ^b^	0.07 ± 0.03 ^a^	0.04 ± 0.005 ^b^
Peak 9	0.34	35.10	0.17 ± 0.02 ^b^	0.25 ± 0.05 ^a^	0.21 ± 0.05 ^a^	0.21 ± 0.02 ^a^	0.23 ± 0.02 ^a^
Peak 10	0.34	37.00	0.09 ± 0.01 ^b^	0.15 ± 0.03 ^a^	0.16 ± 0.01 ^a^	0.12 ± 0.01 ^b^	0.14 ± 0.02 ^a^
Peak 11	0.18	41.90	34.90 ± 0.10 ^a^	33.58 ± 0.05 ^c^	33.59 ± 0.13 ^c^	33.84 ± 0.10 ^b^	33.29 ± 0.01 ^d^
Peak 12	0.18	45.30	39.83 ± 0.14 ^b^	39.77 ± 0.03 ^b^	39.79 ± 0.02 ^b^	40.36 ± 0.02 ^a^	39.26 ± 0.10 ^c^
Total			80.02 ± 0.01 ^b^	80.03 ± 0.01 ^b^	80.05 ± 0.04 ^b^	81.10 ± 0.01 ^a^	78.45 ± 0.01 ^c^

Mean values ± standard deviation in the same row followed by different letters (a–d) are significantly different (Tukey, *p* < 0.05); RT: Retention time; nd: Not detected; Peaks 1–2 were estimated as polysaccharides or high molecular weight carbohydrates (HMWC). Peaks 3–4 were estimated as medium or low molecular weight carbohydrates. Peaks 5–6 were estimated as trisaccharides. Peaks 7–10 were estimated as disaccharides. Peaks 11–12 are monosaccharides.

**Table 4:** Total polyphenol content and multifunctional antioxidant capacity of date fruit powder before and after UVC irradiation for 5, 10, 20, and 40 min.

Sample	Fast-Blue (mg GAE/g dw)	Folin (mg GAE/g dw)	FRAP (mg TE/g dw)	ORAC (µmol TE/g dw)	DPPH EC50 (mg TE/mL)
Untreated	106.17 ± 2.66 ^a^	16.12 ± 0.62 ^a^	146.54 ± 1.84 ^a^	75.60 ± 8.24 ^a^	21.30 ± 0.27 ^b^
5 min	99.14 ± 0.52 ^b^	14.12 ± 0.08 ^b^	88.50 ± 0.12 ^b^	62.74 ± 12.55 ^ab^	58.24 ± 1.11 ^a^
10 min	92.10 ± 1.40 ^c^	13.82 ± 1.03 ^bc^	86.35 ± 0.30 ^c^	61.65 ± 6.88 ^b^	58.27 ± 0.47 ^a^
20 min	73.46 ± 0.17 ^d^	11.01 ± 2.03 ^c^	77.60 ± 0.24 ^d^	60.32 ± 2.05 ^b^	58.73 ± 0.97 ^a^
40 min	104.81 ± 3.32 ^a^	15.28 ± 0.65 ^a^	77.70 ± 0.94 ^d^	64.57 ± 14.45 ^ab^	57.14 ± 0.40 ^a^

Data are mean values ± standard deviation. Values with letters (a–d) were significantly different (Tukey, *p* < 0.05).

## Data Availability

The original contributions presented in the study are included in the article, further inquiries can be directed to the corresponding author.
